# Maternal and paternal age, birth order and interpregnancy interval evaluation for cleft lip-palate

**DOI:** 10.1590/S1808-86942010000100018

**Published:** 2015-10-17

**Authors:** Daniella Reis Barbosa Martelli, Kaliany Wanessa da Cruz, Letízia Monteiro de Barros, Marise Fernandes Silveira, Mário Sérgio Oliveira Swerts, Hercílio Martelli Júnior

**Affiliations:** 1 MSc, Professor of Semiology - State University of Montes Claros - Unimontes; 2 Dentist - Scientific Initiation Scholarship Holder - CNPq; 3 PhD, Professor - University of Alfenas; 4 MSc. Professor - Graduate Program in Health Sciences - State University of Montes Claros -Unimontes; 5 PhD, Professor - University of Alfenas; 6 PhD, Full Professor

**Keywords:** cleft lip, cleft palate, maternal age, paternal age, birth order

## Abstract

Cleft lip and palate (CL/P) are the most common congenital craniofacial anomalies.

**Aim:**

To evaluate environmental risk factors for non-syndromic CL/P in a reference care center in Minas Gerais.

**Materials and Methods:**

we carried out a case-controlled study, assessing 100 children with clefts and 100 children without clinical alterations. The analysis dimensions (age, skin color, gender, fissure classification, maternal and paternal age, birth order and interpregnancy interval), obtained from a questionnaire; and later we build a data base and the analyses were carried out by the SPSS 17.0 software. The results were analyzed with the relative risk for each variable, in order to estimate the odds ratio with a 95% confidence interval, followed by a bivariate and multivariate analysis.

**Results:**

among 200 children, 54% were males and 46% were females. As far as skin color is concerned most were brown, white and black, respectively. Cleft palates were the most common fissures found (54%), followed by lip cleft (30%) and palate cleft (16%).

**Conclusion:**

although with a limited sample, we noticed an association between maternal age and an increased risk for cleft lip and palate; however, paternal age, pregnancy order and interpregnancy interval were not significant.

## INTRODUCTION

Neural tube defects and orofacial fissures are among the most common congenital alterations^1^. Non-syndromic cleft lip and palate (CLP) are the most common anomalies in the skull-facial area (OMIM 119530). In many regions of the world, CLP is more common than the Down syndro-me^1^. Every two minutes, a child with CLP is born in the world, 660 children daily and 235 thousand new cases of fissures are seen annually. With the growth of the world population, we expect 3,200 new cases per year of CLP^2^.

The incidence of CLP varies according to geographic location, race and socio-economic condition^3^. Fogh-Andersen^4^ reported an incidence of 1.5 cases of CLP for every 1,000 births in Denmark, while in other regions the occurrence varied (1–2.69:1,000)^1^,^5^. Recently, Martelli-Júnior et al.^6^ reported an incidence of 1.46 fissures for every 1,000 births, in the state of Minas Gerais, Brazil. Studies show that the Asian population, ancestors of Native Americans and Northern Europeans have a higher incidence of CLP^7^. In contrast, Africans and descendants have a greater incidence of lip fissure^8^.

As far as embryology is concerned, CLPs result from primary defects on the craniofacial fusion which form the primary and secondary palates, in the first trimester of the intrauterine development^9^. These clinical fissures may be classified, having the incisive foramen as anatomical basis, in four groups: pre-incisive foramen or cleft lip (CL), post-incisive foramen fissures or cleft palate (CP), transincisive foramen fissures or cleft lip and palate (CLP) and rare facial fissures^10^.

Together, the CLP make up a heterogeneous group of alterations, having a multifactorial origin, other genetic and environmental factors contribute to their etiology. It is greatly important to identify the etiological factors associated with a disease, because by knowing them it is possible to enhance our understanding of the disease and better develop prevention measures^11^. Among the environmental risk factors for CLP we stress: smoking, maternal and paternal age, alcohol, birth order, interpartum interval and folic acid deficiencies^8^,^11^. The goal of the present study was to evaluate, because of the lack of Brazilian studies, the association or not of environmental risk factors, especially maternal and paternal age, birth order and interpartum interval with CLP.

## MATERIALS AND METHODS

We carried out a case-control study in order to assess the environmental risk factors associated with CLP, in a multiprofessional Reference Service for craniofacial deformities in the state of Minas Gerais, Brazil, between 2006–2008. The population in this study was made up of 200 children with and without non-syndromic CLP from similar socio-economic backgrounds. The group “case” had 100 parents from children (aged between 0 and 12 years), with non-syndromic CLP, diagnosed and being rehabilitated in the aforementioned institution, regardless of gender, skin color, place or country of birth. The “control” group had 100 parents of children in the same age range (between 0 and 12 years); however, without clinical alterations or craniofacial defects, seen at the odontope-diatrics department of the same institution. Both groups had similar socio-economic backgrounds; and also the other inclusion criteria were similar. The aforementioned Reference Center treats exclusively patients from the public health care system (SUS), being certified by the Ministry of Health. From both groups we excluded the parents who did not accept to participate in the study, children with syndromic CLP and parents from consanguineous marriages.

In order to assess the risk factors, in both groups we used an individual instrument (guided questionnaire), encompassing the following analysis aspects: age, skin color, gender, cleft type, parents' ages, birth order and interpartum interval. The questionnaires were deployed always by the same examiners (DRBM and KWC) after being properly trained for the activity. The questionnaire was answered by both groups always after the clinical visit, with the mothers, thus avoiding any harm to the patients in terms of consultation and clinical visit. Each questionnaire was answered in one single visit. Previously, we held a pilot study to assess and check examiners' calibration and the very feasibility of the data collection instrument.

In the “case” group, the non-syndromic CLP cases were classified having the incisive foramen as anatomical reference10 in: (1) CL: including complete or incomplete, uni or bilateral pre-foramen clefts; (2) CLP: including unilateral and bilateral transforaminal clefts and pre and post-foramen clefts; (3) CP: include all the complete and incomplete post-foramen clefts and (4) Others: the rare facial clefts.

After deploying the questionnaires, the information collected was filed in a data bank and analyzed by the SPSS version 17.0 (Chicago, EUA) statistical software. The data was analyzed with relative risk for each variable in order to estimate the odds ratios (OR) with a 95% confidence interval, followed by a bivariate and multivariate analysis. This study was led according to the guidelines established by Ordinance 196/88 from the National Health Council -Ministry of Health, and it was submitted to and approved by the Ethics Committee of the University. Each participant in the study signed a Free and Informed Consent Form.

## RESULTS

In the “case” group, of the 100 children with CLP, 64 (64%) were males and 36 (36%) were females, while in the “control” group, 56 (56%) were females and 44 (44%) males. As far as skin color is concerned, among the cases, 64%, 30% and 6% were brown, white and black, respectively; while among the controls, 45%, 32% and 23% were, brown, white and black, respectively. Table 1 shows, in both groups, the children distribution according to the different ages. We see that among the “cases” there was a prevalence of children between 0 and 3 years, representing 44% in this group; while in the “control” group there was a greater prevalence of children between 7 and 9 years, representing 44% in this group. Thus, when both groups are considered together, we see a predominance of children between 7 and 9 years of age.

**Table 1:** Children from the “case” and “control” groups distributed according to age.

	“Case” Group	“Control” Group
Age range (years)	n	n
0 a 3	44	2
4 a 6	22	35
7 a 9	17	44
10 a 12	17	19

Total	100	100

Table 2 shows the prevalence of CLP seen among the participants of the study, as well as their percentage values in the general distribution and distribution by group. We see that there were no cases of rare clefts in this population. The most common among the clefts studied was the CLP (54%), followed, respectively by cleft lip (30%) and cleft palate (16%). Table 3 shows the distribution of maternal and paternal ages in children with CLP, by age range. We see that both among “cases” and “controls” there was a greater prevalence of mothers between 26 and 35 years. In the age range up to 25 years, the “cases” had a greater number of mothers, when compared to the “controls” (26% and 12%, respectively), and such distribution was inverted in women older than 35 years, where there were more mothers in the “control” group when compared to “cases” (34% and 22%, respectively). As far as paternal age is concerned, we see that it was broken down in intervals up to 40 years and above 40 years. Both in the group “case” and in the “control” group we observed a frequency of up to 40 years, respectively 75% and 70%.

**Table 2:** Type and prevalence of cleft lip and palate, by group and in general.

CLP type	n	Prevalence in the group (%)	Prevalence in general (%)
Cleft palate	16		16
Complete	4	25	4
Incomplete	12	75	12
Cleft lip	30		30
Unilateral Complete	15	50	15
Unilateral Incomplete	14	46,6	14
Bilateral Complete	1	3,3	1
Cleft lip and palate	54		54
Unilateral Complete	36	66,6	36
Bilateral Complete	18	33,3	18

Total	100	100	100

**Table 3:** Distribution of paternal and maternal ages in the “case” and “control” groups.

	“Case” Group		“Control” Group	
Maternal age (years)	n	%	n	%
≤ 25	26	26	12	12
26 to 35	52	52	54	54
> 35	22	22	34	34
Paternal age (years)				
≤ 40	75	75	70	70
> 40	25	25	30	30

Total	100	100	100	100

In relation to the number of pregnancies, in both groups we noticed that among “cases” 39% of the mothers had 1 gestation, 29% (2 gestations) and 15% with 3 gestations. We still found 8% of the mothers who had 4 gestations, 6% with 5 gestations and 3% with 6 gestations. Among “controls”, 2 gestations were more frequent (39%), 3 (24%), 1 (18%) and 4 gestations were less observed (10%), 5 (6%), 8 (2%) and 6 (1%). As far as birth order is concerned, Table 4 shows the distribution both in the “cases” group as well as in the “control” group of children with CLP. We observed that 74% of the children with clefts were born in the first two generations. There were no statistical differences between the groups in relation to birth order (OR: 0.6; 95% CI: 0.34–1.05). All the information associated with the birth order was established considering 1, 2, 3, 4 or more gestations for individuals with CLP and in the “control” group. Category “1” was considered without risk for CLP (OR=1).

**Table 4:** Distribution of birth order in the “case” and “control” groups.

	“Case” Group		“Control” Group		Total	
Birth order	n	%	n	%	n	%
1st child	50	50	37	37	87	43,5
2nd child	24	24	36	36	60	30
3rd child	12	12	17	17	29	14,5
4th child	9	9	5	5	14	7
5th child	5	5	2	2	7	3,5
7th child	0	0	2	2	2	1
10th child	0	0	1	1	1	0,5

Table 5 shows the use of a bivariate analysis in relation to size, maternal and paternal ages and interpartum interval. We have also seen that maternal age showed statistical significance (p<0.05), which was also observed in the logistical regression (multivariate analysis). When the maternal and paternal ages were additionally statistically treated there was no statistical significance (p<0.05).

**Table 5:** Bivariate analysis of the maternal and paternal age and interpartum interval of the “case” and “control” groups.

Variable	Category	“Case”		“Control”		OR	CI-95%	p
	≤ 25	26	26	12	12	1,0		
Maternal age (years)	26 a 35	52	52	54	54	0,42	0,19–0,95	0,037*
	> 35	22	22	34	34	0,29	0,12–0,69	0,006*
	≤ 40	75	75	70	70	1,0		
Paternal age (years)								
	> 40	25	25	30	30	0,70	0,36–1,35	0,284
	1 a 24	20	40	18	28,57	1,0		
Interpartum interval	25 a 48	13	26	16	25,39	0,58	0,22–1,57	0,285
	> 48	17	34	29	46,03	0,53	0,22–1,26	0,152

* statistically significant value (p<0.05).

The interpartum interval was distributed in the following chronological intervals: 1 to 24 months, 25 to 48 months and above 48 months. We did not observe statistical differences between the “cases” and “controls” and the interpartum intervals.

## DISCUSSION

Different epidemiological studies have been carried out in order to assess CLP distribution^12^,^13^. It is accepted that the different types of clefts with distinct distribution and the incidences vary among the different populational groups^14^. Notwithstanding, Orientals, Native Americans, Australian Aborigines and Northern Europeans represent the most affected populations; while Africans and African descendants are more affected by isolate CP8. In the present study, of the 100 clefts assessed, the most commonly found was the CLP, making up 54% of the ones observed. Following the CLP, the CL alone represented 30% and the CP made up 16% of the population studied. A study assessing 126 Brazilian children with non-syndromic CLP showed a prevalence of 2.57 times higher of CLP in males, when compared to females. This same study showed a greater occurrence of CLP followed, respectively of CL and CP alone. Recently, a study carried out in the same institution where this study was held, showed were similar results to the ones hereby presented^16^.

Maternal age is considered a risk factor for numerous chromosomal alterations; however, there is no consensus whether or not it represents a risk factor for CLP. Most of the studies prior to 1970 suggested an association between CLP and maternal age^17^. As of 1970, many papers were published showing conflicting results^17^. A study carried out in California showed that women older than 39 years had twice the risk of having a child with CLP when compared to mothers between 25 and 29 years^18^. Another study with Americans, residents of San Francisco found an association between CP and young women^19^. The epidemiological analysis with a Chinese population showed a relationship between advanced maternal age and bilateral CLP among males and CLP among females^20^. Nonetheless, studies carried out in Canada, Iran, Holland and South America did not show association between maternal age and CLP^17^. The results from the present study showed that, although with a limited population of patients with CLP, maternal age was significant in relation to the occurrence of clefts - using the OR analysis and a 95% confidence interval (Table 5). We noticed that the temporal intervals were from 26 to 35 years and higher than 35 years had reduced risk of having CLP when compared to women with ages lower than 26 years. In a Meta analysis done by Vieira et al.^17^, they did not report any general association between maternal age and CLP. There was a relation between CP and women between 20 and 24 years and above 30 years. One important confounding factor in this study can be the race profile of the populations evaluated. A difficulty in assessing the subjects' social and economic backgrounds, especially in different populations and in different countries; which can explain the differences in the populations studied. In the present study, the population evaluated was from the State of Minas Gerais - a mix of Europeans (mostly Portuguese and Italians), Africans and Native South Americans in a smaller percentage.

Another confounding factor of maternal age is paternal age. It is well known that advanced paternal age (> 40 years) is associated with a risk increase in different diseases, such as achondroplasia, Apert Syndrome and Neurofibromatosis, and a relation with CLP is possible^17^. Association of mutations in MSX1 (muscle segment home-obox) and CLP corroborates the hypothesis of the relation between paternal age and CLP, which has been observed in the Apert syndrome^8^. Hay^21^ and Bille et al.^22^ reported that the risk of CLP with increased maternal age can be reflected in the increase in paternal age. In the present study paternal age did not show statistically significant association with CLP (OR: 0.7; 95% CI: 0.36–1.35) (Table 5). We also did not observe statistical significance when we assessed maternal and paternal ages associated with CLP risk.

There is evidence showing an association between birth order (children of later gestations) and diseases such as: type 1 diabetes, schizophrenia and breast cancer^23^. There is also an association between birth order and congenital heart diseases, neural tube defects and CLP^20^. Nonetheless, there is no consensus whether birth order has any association with CLP^23^. In the present study there was no significant statistical association between these variables (OR: 0.6; 95% CI: 0.34–1.05). Among CLP patients, 74% were born from the first two gestations. Even when the statistical analysis was carried out in the group of patients with CLP, there was no association between birth order and CLP (x2=0.76; p=0.383). One of the first case-control studies carried out to check the association between birth order and CLP was held in 1953, in England, and they did not report correlations between the two conditions^24^. Later on, evaluations carried out in populations from Sri Lanka, France and Iran also did not report associations between birth order and CLP^25^, ^26^, ^27^. On the other hand, investigations carried out in Latin America, Switzerland, USA and China showed positive associations between birth order and CLP^20,^^28,^^29^. Vieira and Orioli^23^, did a Metanalysis on the order of birth and CLP and showed a positive statistical association between the conditions.

Scientific investigations have shown an association between a short interpartum interval (< 6 months) and many alterations, including neural tube defects, congenital heart diseases, low birth weight babies and anemia^11^. Such association has been assigned to a reduction in the levels of folic acid^30^. There are indications that a supplementation with folic acid may have a protective effect for numerous congenital defects, including CLP^31^. Considering these alterations, especially folic acid supplementation, has proved a reduction of 1/3 in the risk of CLP^32^. Reductions in the levels of folate seen in reduced interpartum intervals have been associated to an increased risk of fetal growth restriction^33^. Recently, it was shown that maternal obesity and longer interpartum intervals are associated with CP^34^. In the present investigation, the interpartum periods were classified in three intervals, and we did not observe statistically significant differences among them (Table 5).

As to the pathogenesis of CLP, Vieira^9^ compared this biological event with a jigsaw puzzle with more than 100 pieces and that many genes (between 3 and 14), plus some risk factors involved in the origin of CLP. Thus, although we understand better the participation of genes such as the IRF^6^ (Interferon 6 Regulatory factor), FGF (fi-broblastic growth factor), MSX1 and environmental risk factors, the practical application of such knowledge is still limited. Thus, studies to better understand the action of these agents in animal models and in vitro will enable us to develop more efficient therapeutic tests in the future.

## CONCLUSION

In this case-controlled study we assessed the environmental risk factors associated with non-syndromic CLP and we observed that there was a predominance of CLP, CL and CP alone, respectively. Although with a limited population coming from only one Brazilian state, we noticed an association between maternal age and increased risk for CLP. We also noticed a temporal interval of 26 to 35 years and above 35 years with a reduced risk for CLP compared to women younger than 25 years of age. Notwithstanding, paternal age, birth order and interpartum interval were not statistically significant for the occurrence of CLP.

Acknowledgement: We thank the patients and family members who participated in the study. We appreciate the suggestions from Dr. Antônio Prates Caldeira. We thank the Research Support Foundation of the State of Minas Gerais (Fapemig) and the National Council for Scientific Development (CNPq) (HMJ).
